# Use of Medicines with Unknown Fetal Risk among Parturient Women from the 2004 Pelotas Birth Cohort (Brazil)

**DOI:** 10.1155/2012/257597

**Published:** 2012-12-31

**Authors:** Andréa Dâmaso Bertoldi, Tatiane da Silva Dal Pizzol, Aline Lins Camargo, Aluísio J. D. Barros, Alicia Matijasevich, Iná S. Santos

**Affiliations:** ^1^Programa de Pós-Graduação em Epidemiologia, Universidade Federal de Pelotas, Rua Marechal Deodoro, 1160, 3 Piso, 96020-220 Pelotas, RS, Brazil; ^2^Programa de Pós-Graduação em Epidemiologia, Universidade Federal do Rio Grande do Sul, Rua Ramiro Barcelos, 2400, 2 Andar, 90035-003 Porto Alegre, RS, Brazil; ^3^Departamento de Ciências Básicas da Saúde, Universidade Federal de Ciências da Saúde de Porto Alegre, Rua Sarmento Leite, 245, 90050-170, Porto Alegre, RS, Brazil

## Abstract

*Background*. To estimate the exposure to medicines with unknown fetal risk during pregnancy and to analyze the maternal characteristics associated with it. *Methods*. A questionnaire was administered to 4,189 mothers of children belonging to the 2004 Pelotas (Brazil) birth cohort study about use of any medicine during gestation. We evaluated the associations between use of medicines with unknown fetal risk and the independent variables through logistic regression models. Unknown fetal risk was defined as medicines in which studies in animals have revealed adverse effects on the fetus, and no controlled studies in women, or studies in women and animals, are available. *Results*. Out of the 4,189 women, 52.5% used at least one medicine from unknown fetal risk. Use of these medicines was associated with white skin color, high schooling, high income, six or more antenatal care consultations, hospital admission during pregnancy, and morbidity during gestation. *Conclusion*. The use of unknown fetal risk medicines is high, suggesting that their use must be addressed with caution with the aim of restricting their use to cases in which the benefits are greater than the potential risks.

## 1. Background

Studies have demonstrated high prevalence of medicine use during pregnancy. The exposure to at least one medicineduring pregnancy is most often exceeding 70% [1–10]. A recent systematic review, which evaluated medicine use prescribed during pregnancy in developed countries, showed that 27 to 93% of the pregnant women received at least one prescription of medicine, excluding vitamins and minerals [11]. 

In many situations the use may be unnecessary and even dangerous for the fetus, although one should consider that in most cases the use of medicines to treat chronic conditions and problems associated with pregnancy can be vital to the health and well-being of the woman and the offspring [12, 13]. 

Prescription of medicines during pregnancy is challenging for doctors because they need to balance the benefits of the treatment against possible damages to the fetus [14]. Most studies evaluating the risk that medicines pose to the fetus used the US Food and Drug Administration (FDA) classification [15]. Such studies have shown that exposure to category A medicines (considered safe during pregnancy) occurred in 49% to 100% of the participating women [5, 7, 16, 17]. Medicines with evidence of fetal risk (category D and X) were used by less than 12% of pregnant women [1, 5, 7, 17–20], with the exception of a study conducted in France, in which the percentage of exposure to category D drugs was 59% [16]. Many studies focused on the factors associated with the use of products from categories D or X [18, 19, 21, 22]. 

The most challenging category of medicines in this case, however, is the one in which the risk to the fetus is unknown, classified by the FDA in Category C. This group represents an important fraction when considering the quantity of prescribed drugs and the number of exposed women. Studies indicate that the use of unknown fetal risk medicines during pregnancy ranges from 19% to 40% [1, 5, 6, 23]. The present study aimed to verify the prevalence of use of medicines with unknown fetal risk during pregnancy and to analyze maternal characteristics associated with this outcome.

## 2. Methods

In 2004, the third birth cohort in the city of Pelotas, southern Brazil was initiated. In this study, all mothers of babies born in the city's five maternities (99% of all births) were interviewed within the first 24 hours that succeeded childbirth. A standardized precoded questionnaire was applied, including questions about the use of any medicine during pregnancy and the initiation month and termination of this usage. The question about medicine use was: “Please, let us know the name of all medicines that you have used during pregnancy. Please remember to consider those used for nausea, heartburn, anemia, treatment of urinary tract infection, vaginal infection, high blood pressure, or diabetes.” Further details on the methodology of the cohort can be found elsewhere [24].

 Medicines with unknown fetal risk were classified as those in which studies in animals have revealed adverse effects on the fetus (teratogenic or embryocidal or other), and there are no controlled studies in women or studies in women and animals are not available. 

We decided to use criteria adopted by the FDA [15] for the category C, in order to enable possible comparisons since this is the most frequent classification found in different studies. Medicines showing evidence about risk, leading to damage or safety of the fetus, were classified as medicines with known fetal risk. 

 Firstly, the drug monograph was queried in the DRUGDEX System [25], followed by consultation of the Guide to Fetal and Neonatal Risk by Briggs et al. [26]. When the information was not located in any of the two sources, a search in PubMed was performed by intersecting the drug name with keywords related to pregnancy and teratogenicity/adverse effects. Medicines without any information about the teratogenic risk were classified as unknown fetal risk [26], in accordance with previous studies [5, 23]. Medicines were also classified as unknown fetal risk when information was available on the risk of the therapeutic group, but not the medicine itself (e.g., benzodiazepines versus bromazepam). When information was available on the risk of the active substance, but not for the salts, the classification of the main element was adopted (e.g., when there was information for iron, but not for the iron polymaltose). In the case of medicines for which the risk category changes depending on the dose (e.g., ferrous sulphate) the classification was based on the most common prescribed dose. When drugs were mentioned by commercial name, the dosage use indicated by their manufacturer label was considered to establish the risk. 

Regarding the association of medicines, the evidence of fetal risk was investigated to each individual component of the formulation. The formulation was classified as medicine with known fetal risk when at least one of the components of the formulation was identified with evidence of risk. 

Statistical analyses were performed using SPSS version 18.0. We first analyzed the association between medicine use in general and the use of unknown fetal risk medicines. We then repeated the analyses after excluding vitamins and iron supplements. The variables of interest were: trimester in which the medicine was used, age of the mother, skin color, years of schooling, marital status, per capita income, birth order, number of prenatal consultations, gestational week of the first prenatal consultation, hospitalization during pregnancy, and the presence of the following self reported clinical conditions: hypertension, diabetes, depression, anemia, threatened abortion, threatened premature birth, and urinary tract infection. Chi-square tests were used for comparing proportions. Logistic regression was used for adjusted analysis, adjusting all the variables of interest. A significance level of 5% was employed.

The study was approved by the School of Medicine Ethics Committee of the Federal University of Pelotas. 

## 3. Results

Out of a total of 4,189 interviewed pregnant women, 92.7% reported the use of some medicine during pregnancy, totaling the use of 11,425 medicines. The number of medicines used during gestation ranged from 1 to 10 medicines by pregnant women with an average of 2.9 (SD 1.6). Considering the pregnancy trimesters, the average number of medicines used was 1.3 (SD 0.6), 1.4 (SD 0.7), and 1.7 (SD 0.9) during the first, second, and third trimesters, respectively. Among the women who reported the use of medicines, 61.2% used one or two medicines, 28.7%, three or four medicines and 10.3%, five or more. If vitamins and iron are excluded from the analyses, 1,829 pregnant women (43.7%) reported using at least one medicine, adding up to the use of 7,860 medicines.

Out of all medicines used, 38.9% were identified as unknown fetal risk. More than half of all mothers used at least one product with unknown fetal risk (52.5%).

Table 1 summarizes the results for the trimester of the first usage for each medicine as well as the period of their use. The number of medicines with usage initiated in each trimester was very homogeneous (around 30% for each trimester) and the largest proportion of medicines was used exclusively in the third trimester. Only 5.1% of the medicines used in the first trimester were also used during the second trimester and 14.6% of the medicines were used during the three trimesters.

The most commonly used medicines with unknown fetal risk are presented in Table 2, by the gestational trimester of use. Multivitamins and the association between scopolamine butyl bromide and dipirone accounted for more than 50% of the medicines used in any time during pregnancy.

Table 3 presents demographic, socioeconomic, and health characteristics of the mothers during pregnancy. Data on the total prevalence of use of medicines (92.7%) and on the use of medicines with unknown fetal risk (52.5%) according to such variables are presented. The following variables remained associated to the use of medicines with unknown fetal risk after adjusted analyzes: white skin color, education and per capita income (in direct proportion to use), six or more prenatal consultations, hospitalization, and morbidity during the pregnancy (depression, anemia, threat of abortion, threat of premature labor, and urinary infection).

Table 4 presents the same analysis conducted in Table 3, but excluding vitamin and iron supplements. The total prevalence of use of medicines excluding vitamins and iron was 43.7%. The prevalence of use of medicines with unknown fetal risk was 25.6%. The following variables were associated with the use of medicines with unknown fetal risk in the adjusted analyses: per capita income, hospitalization, threat of abortion, and threat of premature labor.

In Table 5 we analyzed only women who reported to have diabetes or hypertension. Medicines used for treating diabetes were insulin and metformin, and, for hypertension the most frequent medicine used was methyldopa followed by others with low frequencies classified as “unknown risk”. The prevalence of use of antidiabetics was 13.8% and the prevalence of use of antihypertensives medication was 25.8%. Among diabetic women, no antidiabetic medicine of unknown fetal risk was reported to be used, whereas 38% of the other medicines used were classified as of unknown fetal risk. Among hypertensive women, 9.6% of the hypertension medication used was classified as of unknown fetal risk, whereas 41.3% of the other medicines used were classified as such. 

## 4. Discussion

In this study the frequency of exposure to medicines by gestational trimesters, the identification of the medicines with unknown information about fetal risk, and the main factors associated to these medicines were examined. Some features of this study are distinctive from other similar studies in the literature: the inclusion of prescription and over-the-counter medicines, since the majority of studies do not consider medicines used for self-medication or those prescribed during hospitalizations [16, 27], and collection of data by interview with the mothers instead of using secondary data. Data presented here are directly generalizable to the population of the city, because virtually all mothers who gave birth in 2004 were included. Findings are also likely valid for other areas of Brazil, which similarly to Pelotas have huge socioeconomic disparities leading to inequities in access to health care. Therefore, we believe data may be extrapolated to other municipalities with similar characteristics thus contributing to the improvement of knowledge regarding factors associated to medicine use with unknown risk during pregnancy in Brazil. Extrapolating our findings to other low and middle income countries is likely misleading due to huge heterogeneity in pharmaceutical policies. 

The prevalence of the use of at least one medicine during pregnancy was high; however, it is consistent with other studies conducted in Brazil with prevalence over 90% [4, 9]. This usage is also quite high in other countries. However, most studies investigated only the use of prescription medicines [5, 16, 18, 28], potentially resulting in estimates that are lower than the prevalence identified in this study. Other fact to be considered is the type of question used in our study. We opted to use an open-ended question followed by selected indication-orientation (the question used in our study is described in the methods section). de Jong-van den Berg and colleagues [29] evaluated the influence of the type of questions used in interviews about medicine use in pregnancy, and concluded that the prevalence of medicine use increases considerable when the question about medicine use includes indication-orientation and drug-orientation to the open-ended questioning. The authors also concluded that these higher prevalence figures are more comparable with pharmacy records.

It was observed that the prevalence of medicine use by pregnant women exceeds the prevalence for women in general [30–32]. In studies carried out in Brazil to evaluate medicine utilization in the general population, women in reproductive age (18 to 49 years old) presented prevalence of medicine use from 42% [33] to 60.4% [30]. In a European study it was found a slightly higher prevalence (68.8%), considering women aged 25 to 44 years old. [32]. The use of medicines indicated during the gestational period, such as vitamins and iron derivatives, can explain these differences, which is attenuated when these groups of medicines are excluded from the analysis. 

The present study reports the gestational trimester in which the medicine use started, while available data in the literature reports only whether the pregnant women were exposed to medicines in different periods [3, 5, 8, 10, 34]. The largest number of used medicines was reported as first used during the second trimester (35.6%) and a slightly smaller proportion (29.9%) was reported as first used during the first trimester, which may indicate an issue of concern about exposure to medicines in early pregnancy where the risks to the fetus are known to be higher [35]. 

Only 14.6% of the medicines were used throughout the entire gestational period, indicating that situations in which the continued use of medicines is necessary are few. However, this percentage does not necessarily indicate that medications are unnecessary—the medication may be warranted, but women may not continue use, due to nonadherence or concerns about use after realizing that they are pregnant.

 In addition, pregnant women are usually younger and healthier that women in general, which leads to the use of medicines for acute situations, such as infection, the control of symptoms commonly associated with the pregnancy itself (such as heartburn) or prophylactically indicated during the pregnancy (such as folic acid). 

Most studies classifying medicines in risk categories used the FDA criteria [1, 4–7, 16, 18–21, 26, 28, 36]. Some studies excluded vitamins and minerals [1, 18] from the analyses while others evaluated the use of the medicines in part of the gestation period (up to the gestational age at the time of the interview) [23, 36]. Only studies that evaluated the use of medicines throughout the whole gestational period and included vitamins and minerals were selected for the purpose of comparisons with our data [4–7, 16, 18, 21, 28].

The frequency of the use of medicines with unknown fetal risk (38.9%) is consistent with a study conducted with parturients interviewed after childbirth in a hospital from another Brazilian city (42.4%) [4], however, higher than the reported frequency in a study conducted in a city in southern Brazil (24.5%) [6] and in Ethiopia (15.2%) [7]. 

The prevalence of women exposed to the medicines with unknown fetal risk (52.5%) was higher than that observed in most studies (14.7 to 19%) [5, 7, 28] but lower than the one reported in the study by Lacroix (85.0%) [16]. The study by Lacroix presents the peculiarity of inclusion of products that generally were not included in other studies, such as homeopathic remedies [37], thus leading to its elevated percentages. Other studies do not mention having investigated the primary literature about teratogenic risk, which may have led to an underestimated classification of many medicines in this group [5, 7, 18, 21, 28]. According to the FDA's recommendations, the use of these medicines should only occur when the potential benefits justify the potential risks to the fetus. As a consequence, the uncertainty about the risks associated with this category can generate doubts and anxiety in doctors and pregnant women. However, medical information justifying the use of these medicines by each pregnant woman is not available for analysis, which would allow a better assessment of the impact from the use of these medicines.

Formulations with multiple vitamins stood out in the analyses of the medicines with unknown fetal risk during pregnancy. The risk of some vitamins during gestation is dose-dependent. The multivitamin's components were analyzed individually and classified according to their dosage in the formulation. According to Briggs et al. [26], the following vitamins, in concentrations higher than the ones described as follows, present unknown fetal risk: vitamin B1 (1.5 mg), vitamin B2 (1.6 mg), vitamin B5 (10 mg), vitamin B12 (2.2–2.6 *μ*g), vitamin C (70 mg), vitamin E (10 mg), and nicotinamide (17 mg). The following vitamins show positive evidence of fetal risk in concentrations higher than 400 IU and 8,000 IU/day, respectively, for Vitamin D (and D3) and vitamin A. 

The use of multivitamins during pregnancy is often suggested as an intervention aimed at improving maternal and fetal health. However, substantive evidence regarding the effectiveness of multiple-micronutrient supplements during pregnancy is not available. Its use during the prenatal period is associated with controversial results in the literature which points to positive results [38] and lack of effect [39] in terms of the benefit of multivitamins in the outcome of low birth weight. There is not sufficient evidence for other relevant clinical outcomes. Therefore, the use of this type of medicine, advocated in the obstetric practice, should be assessed considering the different existing formulations in the market and acknowledging that, depending on the dose present in the formulation its use is only justified if the potential benefits overcome the potential risks for the fetus.

The analysis of the variables potentially related to a greater use of medicines with unknown fetal risk was also performed for the use of all medicines during pregnancy. White women with higher education and greater income used more medicines with unknown fetal risk. In general, higher education alone was associated with greater use of medicines, which is in agreement with a study conducted in France and others in several Brazilian cities [4, 23, 36, 40]. Primiparous status increases the proportion of the use of medicines (excluding medicines with unknown fetal risk) in this study and other studies in Brazil [23, 36]. Mothers who had a greater number of prenatal consultations used more medicines in general and specifically medicines with unknown fetal risk. However, the use of medicines is not associated to the initiation of prenatal care occurring either before or after the 20th week of gestation. In the study by Guerra et al., prenatal care in the 1st trimester was associated with a higher use of medicines [36].

Generally speaking, the presence of morbidity was associated to an increased risk of the use of medicines in general and also medicines with unknown fetal risk. This was verified among mothers who were hospitalized during the pregnancy and those who presented depression, anemia, threat of premature birth and urinary tract infection. A greater risk of the use of medicines with unknown fetal risk was not observed specifically by the mothers suffering from high blood pressure since, methyldopa was among one of the most used antihypertensive medications by pregnant women. Methyldopa has not been confirmed to present a risk to the embryo or fetus. The increased use of medicines among mothers with chronic diseases was also verified in studies by Costa da Fonseca et al. and Guerra et al. [4, 36].

In order to have some information about use of medicines to treat chronic diseases in pregnancy, we analyzed pregnant women with diabetes and hypertension due to the high burden of disease caused by these two illnesses in Brazil. It was observed that all antidiabetics and most antihypertensive products are of known risk, with only a small percentage of antihypertensive medicines classified as unknown fetal risk. This might be explained by more rigorous prescription patterns for treating these two diseases due to their high burden. 

It was possible to identify that vitamins and iron supplements play a key role at increasing the prevalence of use of medicines with unknown fetal risk in pregnancy. Another issue to be considered is that due to the low cost of iron supplements, these products are widely used by all socioeconomic groups, and therefore, exclusion of them from the analyses maximizes the differences between low and high income women. The association of number of antenatal consultations and use of medicines was also no longer significant when vitamin and iron supplements were excluded from the analysis. The use of such products is more common among low risk pregnant women and those with higher number of antenatal care consultations, because the prescription of antianemic products is highly prevalent in Brazil despite some controversies in the literature [41].

We opted for the use of logistic regression in order to maintain comparability with most of the previous published studies in the subject. However, it should be noted that, because the outcome “use of medicines” has high prevalence, the odds ratios estimated by logistic regression are well above their respective prevalence ratios. 

The absence of a risk assessment in accordance with the medicine doses used by mothers is a common problem in most studies including this one. It is known that teratogenic and fetotoxic effects are usually dependent on dosage and the length of usage time [37]. The fact that the investigation about the use of medicines occurred in a single moment (at the interview time), after childbirth, can lead to recall errors as a function of the long period investigated (nine months). In addition to this, the fact that the mothers did not have the packaging of the used medicines at the time of the interview, which often made it impossible for the identification of the exact name of the medicine used and consequent inability to assign it in the proper risk category due to its unknown composition must also be considered in the overall analyses.

The risk evaluation by the FDA and other similar classifications has been widely criticized, especially as they lead to an incorrect impression that the risk increases from A to X and that drugs from the same category present the same potential risk. Further, the system does not address potential developmental adverse events on the basis of expected incidence, severity, or reversibility, nor whether there are degrees of risk based on the dose, duration, frequency, route, or gestational timing of exposure to a given product. The FDA was led to propose, in 2008, a review to present information about medicine use during pregnancy included in the package insert of the medicines. This proposal about the new regulation has been discussed, but until now the new labeling requirements have not yet been initiated [26, 42, 43]. 

The FDA initiative to revise this classification contributes to an important and necessary reflection about the safety of medicines used during gestation, considering that the classification adopted for many years confounded the prescribers in the moment to decide about the use or not of some medicines. The majority of the medicines do not clearly state the risk or safety associated with the use of the product. This fact lead the category C to an uncertain category, where data available do not state whether the medicines are safe or not. 

## 5. Conclusion

Cohort studies aimed at the measurement of the consequences of the use of medicines during pregnancy are needed. For the advancement of knowledge in this subject, the evaluation of dosages and duration of the use of each medicine by the mothers during pregnancy should be correlated to the health outcomes of the mother and newborn in different phases of development. Health professionals should be frequently educated on relationships between teratogenic agents and maternal and fetal health in order to make appropriate recommendations regarding the use of some medicines based on the best information available and not solely based on classifications that are subject to significant errors, especially for taking into account a short term follow-up of the offspring. Little is known about long term consequences for the offspring and for that reason these classification systems have limitations.

In addition, regulatory changes obligating pharmaceutical companies to include more detailed information about the risks associated with the use of medicines and health professionals' training are needed, particularly during pregnancy, therefore empowering health professionals to prescribe effective and safe medicines to patients. 

## Figures and Tables

**Table 1:** Medicines used by the mothers participating in the 2004 Birth Cohort by the gestational trimester and percentages of mothers who used at least one medicine in the respective periods. Birth Cohort of Pelotas, RS 2004.

Trimester	Medicines (*n* = 11,425)		Mothers (*n* = 3,883)	
	*n*	%	*n*	%
Start of the use of medicines^a^				
1st	3,241	29.9	1,909	50.0
2nd	3,847	35.6	2,377	62.2
3rd	3,738	34.5	2,260	59.1

Total	10,826	100.0	3,821^c^	^ e^

Period of the use of medicines^b^				
1st	1,093	10.1	848	22.2
2nd	1,884	17.5	1,366	35.8
3rd	3,734	34.7	2,259	59.2
1st and 2nd	545	5.1	467	12.2
2nd and 3rd	1,939	18.0	1,504	39.4
1st, 2nd and 3rd	1,575	14.6	1,170	30.7

Total	10,770	100.0	3,814^d^	^ e^

^a^599 missing; ^b^655 missing; ^c^62 missing; ^d^69 missing; ^e^the percentages do not add up to 100% since each mother who used any medicine could have used more than one medicine in the same period or in different periods.

**Table 2:** Most used medicines by the mothers participating in the 2004 Birth Cohort classified as unknown fetal risk, by the gestational trimester of medicine use. Birth Cohort of Pelotas, RS 2004.

				Trimester of use^a^			
Medicines used at any time during pregnancy	Total (*N* = 3,505)	1st (*N* = 292)	2nd (*N* = 527)	3rd (*N* = 1,236)	1st and 2nd (*N* = 160)	2nd and 3rd (*N* = 764)	1st, 2nd, and 3rd (*N* = 526)
	%	%	%	%	%	%	%
Multivitamins	31.2	17.4	20.0	26.6	35.9	46.8	44.8
Scopolamine Butylbromide + dipyrone	27.7	37.6	31.7	32.0	23.7	19.6	21.7
Aluminium hydroxide^b^	7.4	4.5	4.6	3.9	5.3	8.9	6.5
Piperidolate + hesperidine + ascorbic acid	6.5	12.0	6.4	5.1	16.8	6.1	7.2
Nystatin	5.2	7.4	11.5	7.7	0.8	1.3	0.7
Sodium bicarbonate^b^	3.3	2.9	1.1	0.8	0.8	2.7	4.8
Ferrous sulphate + ascorbic acid + folic acid	3.0	2.5	1.6	0.8	4.6	4.1	8.7
Salbutamol	2.6	0.4	2.3	5.2	0.0	2.0	1.3
Isoxsuprine	2.2	1.2	2.5	3.5	1.5	2.1	0.7
Miconazole	1.9	1.7	4.4	2.8	0.0	1.1	0.0
Ascorbic acid	1.6	2.5	0.9	1.9	0.8	1.8	1.3
Diclofenac	1.4	2.9	1.8	1.6	3.8	0.4	0.4
Norfloxacin	1.2	0.8	3.0	1.8	0.0	0.1	0.2
Dimethicone	0.8	0.0	0.7	0.8	1.5	0.9	1.1
Terconazole	0.8	0.8	3.4	0.8	0.8	0.0	0.0
Promethazine	0.8	0.4	0.9	1.3	2.3	0.4	0.4
Betamethasone	0.6	0.0	0.5	1.4	0.0	0.3	0.2
Trimethoprim-sulfamethoxazole	0.6	1.7	1.1	0.5	0.0	0.4	0.0
Pipemidic acid	0.6	0.8	1.6	0.8	0.0	0.1	0.0
Fluoxetine	0.6	2.5	0.0	0.5	1.5	0.7	0.0
Others	8.9	17.1	17.3	20.2	18.1	7.7	12.5

^a^There is no information about in which gestational trimester was the use of 177 medicines classified as unknown fetal risk.

^b^Medicine used individually or in association.

**Table 3:** Description of the sample and the health conditions of the mothers participating in the 2004 Birth Cohort (*N*), prevalence (*P*%) and adjusted analyses of the use of medicines during pregnancy considering all medicines and the medicines with unknown fetal risk used. Birth Cohort of Pelotas, RS 2004.

	*N*	Use of medicines (total)			Use of medicines with unknown fetal risk		
Characteristic			Adjusted analyses			Adjusted analyses	
		*P*%	PR (IC 95%)	*P* value	*P*%	PR (IC 95%)	*P* value
Total of participants	4,189	92.7	—	—	52.5	—	—
Age				0,062			0.076
<20	796	90.8	1.00		43.6	1.00	
20 to 29	2,085	93.1	1.58 (1.03–2.43)		53.2	1.09 (0.90–1.32)	
30 to 39	1,170	92.9	2.01 (1.19–3.38)		56.9	1.31 (1.05–1.64)	
40 or more	136	94.1	2.35 (0.83–6.62)		55.1	1.18 (0.76–1.84)	
Skin color				0,08			<0.001
White	2,555	93.8	1.31 (0.97–1.78)		57.7	1.36 (1.17–1.58)	
Non-white	1,586	90.9	1.00		44.1	1.00	
Education in years				0.026			<0.001
0 to 4	647	87.3	1.00		32.8	1.00	
5 to 8	1,711	91.6	1.50 (1.02–2.23)		47.0	1.70 (1.35–2.13)	
9 to 11	1,396	95.1	1.90 (1.19–3.03)		62.7	2.57 (2.01–3.30)	
12 or more	435	97.2	2.66 (1.22–5.79)		70.6	2.74 (1.95–3.85)	
Marital status				0.064			0.92
With the baby's father	3,502	93.1	1.44 (0.98–2.11)		53.5	0.99 (0.81–1.21)	
Without	687	90.5	1.00		47.0	1.00	
Income per capita				0.09			<0.001
1st quintile	830	88.4	1.00		38.2	1.00	
2nd quintile	834	89.2	0.83 (0.55–1.25)		41.7	1.03 (0.82–1,30)	
3rd quintile	886	94.2	1,48 (0.93–2.36)		52.7	1.29 (1.03–1.62)	
4th quintile	849	94.7	1.11 (0.68–1.82)		61.1	1.79 (1.41–2.27)	
5th quintile	790	96.8	1.55 (0.81–2.95)		69.2	1.94 (1.47–2.55)	
Parity				0.025			0.52
primipara	1,658	94.8	2.45 (1.34–4.46)		55.9	1.12 (0.81–1.56)	
1 or 2	1,086	93.0	1.65 (0.96–2.82)		54.8	1.06 (0.78–1.45)	
3 or 4	1,026	91.6	1.36 (0.83–2.23)		48.6	0.95 (0.70–1.27)	
5 or more	419	86.2	1.00		42.2	1.00	
Prenatal consultation				0.004			0.001
<6	689	86.6	1.00		36.0	1.00	
6 or more	3,250	94.9	1.65 (1.18–2.32)		56.8	1.39 (1.14–1.70)	
Start of prenatal care				0.70			0.32
Before the 20th week	3,765	94.0	1.10 (0.68–1.80)		54.8	1.18 (0.85–1.64)	
After the 20th week	283	84.8	1.00		31.4	1.00	
Hospitalization							
No	3,722	91.9	1.00	0.002	50.9	1.00	0.05
Yes	467	98.9	6.50 (1.99–21.21)		64.7	1.28 (1.00–1.62)	
High Blood Pressure				0.012			0.87
No	3,189	92.3	1.00		52.6	1.00	
Yes	992	94.0	1.60 (1.11–2.31)		52.3	0.90 (0.59–1.37)	
Diabetes				0.76			0.63
No	4,062	92.6	1.00		52.4	1.00	
Yes	124	96.0	0.86 (0.32–2.30)		56.4	0.90 (0.59–1.37)	
Depression				0.034			<0.001
No	3,138	92.1	1.00		51.0	1.00	
Yes	1049	94.3	1.49 (1.03–2.16)		56.9	1.38 (1.16–1.64)	
Anemia				<0.001			<0.001
No	1,402	83.0	1.00		42.7	1.00	
Yes	2756	97.8	10.79 (7.68–15.16)		57.7	1.53 (1.31–1.78)	
Threat of abortion				0.15			<0.001
No	3,736	92.2	1.00		50.0	1.00	
Yes	449	96.4	1.55 (0.85–2.83)		73.0	2.40 (1.84–3.11)	
Threat of premature labor				<0.001			<0.001
Não	3,416	91.7	1.00		48.0	1.00	
Sim	771	97.0	2.72 (1.57–4.73)		72.5	2.65 (2.15–3.27)	
Urinary Infection				<0.001			0.012
Não	2,623	89,8	1.00		52.5	1.00	
Sim	1,552	97.7	5.53 (3.58–8.54)		52.5	0.82 (0.71–0.96)	

*Chi square test; **1st quintile relates to the most deprived quintile ***PR: prevalence ratio.

**Table 4:** Description of the sample and the health conditions of the mothers participating in the 2004 Birth Cohort (*N*), prevalence (*P*%) and adjusted analyses of the use of medicines during pregnancy considering all medicines and the medicines with unknown fetal risk used, excluding vitamin and iron supplements. Birth Cohort of Pelotas, RS 2004.

		Use of medicines (total)			Use of medicines with unknow fetal risk		
Characteristic	*N*	*P*	Adjusted analyses		*P*	Adjusted analyses	
		%	PR (IC 95%)	*P* value	%	PR (IC 95%)	*P* value
Total of participants	4,189	43.7	—	—	25.6	—	—
Age				0.275			0.883
<20	796	38.8	1.00		24.5	1.00	
20 to 29	2,085	44.6	1.19 (0.98–1.44)		26.1	0.98 (0.78–1.23)	
30 to 39	1,170	44.8	1.21 (0.98–1.50)		25.7	0.93 (0.70–1.23)	
40 or more	136	47.8	1.25 (0.82–1.88)		23.5	0.85 (0.51–1.40)	
Skin color				0.038			0.154
White	2,555	45.8	1.17 (1.01–1.35)		27.0	1.12 (0.96–1.32)	
Non-white	1,586	40.2	1.00		23.1	1.00	
Education in years				0.002			0.075
0 to 4	647	40.6	1.00		19.8	1.00	
5 to 8	1,711	40.6	1.09 (0.88–1.35)		25.0	1.28 (1.02–1.61)	
9 to 11	1,396	47.9	1.42 (1.12–1.79)		28.2	1.39 (1.08–1.78)	
12 or more	435	46.9	1.56 (1.14–2.13)		28.5	1.36 (1.00–1.89)	
Marital status				0.615			0.331
With the baby's father	3,502	44.2	1.05 (0.86–1.29)		26.0	1.11 (0.90–1.39)	
Without	687	41.0	1.00		23.3	1.00	
Income per capita				0.009			0.020
1st quintile	830	39.0	1.00		20.5	1.00	
2nd quintile	834	40.3	1.03 (0.83–1.28)		24.8	1.26 (1.00–1.59)	
3rd quintile	886	45.8	1.37 (1.10–1.70)		24.8	1.21 (1.00–1.53)	
4th quintile	849	46.5	1.37 (1.09–1.72)		28.5	1.45 (1.14–1.84)	
5th quintile	790	46.6	1.30 (1.01–1.68)		29.5	1.48 (1.14–1.92)	
Parity				0.991			0.830
primipara	1,658	43.5	0.99 (0.72–1.37)		26.5	0.94 (0.67–1.32)	
1 or 2	1,086	44.5	1.01 (0.75–1.37)		25.9	0.91 (0.66–1.26)	
3 or 4	1,026	44.4	0.98 (0.74–1.30)		24.5	0.88 (0.65–1.19)	
5 or more	419	40.3	1.00		23.9	1.00	
Prenatal consultation				0.747			0.277
<6	689	42.8	1.00		24.2	1.00	
6 or more	3,250	43.9	0.96 (0.77–1.20)		26.2	0.88 (0.70–1.11)	
Start of prenatal care				0.861			0.517
Before the 20th week	3,765	43.7	0.97 (0.71–1.33)		26.2	1.12 (0.80–1.58)	
After the 20th week	283	40.6	1.00		20.8	1.00	
Hospitalization				<0.001			<0.001
No	3,722	42.1	1.00		24.3	1.00	
Yes	467	56.3	1.51 (1.20–1.89)		35.5	1.48 (1.19–1.84)	
High Blood Pressure				<0.001			0.305
No	3,189	40.9	1.00		25.1	1.00	
Yes	992	52.5	1.62 (1.38–1.90)		27.2	1.10 (0.92–1.31)	
Diabetes				0.995			0.390
No	4,062	43.5	1.00		25.4	1.00	
Yes	124	50.8	1.00 (0.67–1.50)		32.3	1.20 (0.79–1.81)	
Depression				<0.001			0.066
No	3,138	41.5	1.00		24.8	1.00	
Yes	1049	50.1	1.45 (1.24–1.70)		28.1	1.18 (0.99–1.40)	
Anemia				<0.001			0.366
No	1,402	64.4	1.00		24.2	1.00	
Yes	2756	32.9	0.23 (0.20–0.26)		26.4	1.08 (0.92–1.26)	
Threat of abortion				0.003			<0.001
No	3,736	42.3	1.00		24.4	1.00	
Yes	449	54.6	1.39 (1.11–1.73)		35.6	1.48 (1.19–1.83)	
Threat of premature labor				<0.001			<0.001
Não	3,416	41.0	1.00		23.5	1.00	
Sim	771	55.3	1.46 (1.22–1.76)		34.9	1.50 (1.25–1.80)	
Urinary Infection				<0.001			0.434
Não	2,623	38.6	1.00		24.6	1.00	
Sim	1,552	52.2	2.10 (1.81–2.42)		27.2	1.06 (0.91–1.24)	

*Chi square test; **1st quintile relates to the most deprived quintile, ***PR: prevalence ratio.

**Table 5:** Medicine use among hypertensive and diabetic women. Birth Cohort of Pelotas, RS 2004.

Women with diabetes (*n* = 124)*				Women with hypertension (*n* = 992)**			
Medicines	*N*	%	% with unknown risk	Medicines	*N*	%	% with unknown risk
Antidiabetics	15	13.8	—	Antihypertensives	219	25.8	9.6
Other	94	86.2	38.0	Other	630	74.2	41.3

*Casos válidos: 109 mulheres com informação sobre o nome dos medicamentos.

**Casos válidos: 849 mulheres com informação sobre o nome dos medicamentos.
